# Chemosensing on Miniaturized Plasmonic Substrates

**DOI:** 10.3390/mi12030275

**Published:** 2021-03-06

**Authors:** Pengcheng Wang, Rodica Elena Ionescu

**Affiliations:** Laboratoire Lumière, Nanomatériaux et Nanotechnologies (L2n), CNRS ERL 7004, Université de Technologie de Troyes, 12 Rue Marie Curie CS 42060, 10004 Troyes CEDEX, France; pengcheng.wang@utt.fr

**Keywords:** annealed ITO/AuNPs, small-sized coverslips, plasmonic platforms, BPE sensing

## Abstract

Round, small-sized coverslips were coated for the first time with thin layers of indium tin oxide (ITO, 10–40 nm)/gold (Au, 2–8 nm) and annealed at 550 °C for several hours. The resulting nanostructures on miniaturized substrates were further optimized for the localized surface plasmon resonance (LSPR) chemosensing of a model molecule—1,2-bis-(4-ppyridyl)-ethene (BPE)—with a detection limit of 10^−12^ M BPE in an aqueous solution. All the fabrication steps of plasmonic-annealed platforms were characterized using scanning electron microscopy (SEM) and atomic force microscopy (AFM).

## 1. Introduction

The topic of surface plasmons has received considerable attention from physicists, chemists, biologists and material scientists for a wide range of applications in photonics and optoelectronic devices, such as surface-enhanced Raman scattering (SERS), photothermal-enhanced fluorescence, optical sensing and biomedicine, due to their potential optical properties. Localized surface plasmon (LSPR), which is an optical phenomenon, occurs when the resonance frequency of the incident light (incident electromagnetic field) corresponds with the free-electron oscillation frequency of conductive nanoparticles (NPs), which are much smaller than the wavelength of the incident light [1]. Such interaction induces an intense and highly localized (surrounding the conductive NPs) electromagnetic (EM) field, which makes NPs sensitive to small changes in the local refractive index (RI) [2]. For instance, organic molecules and biomolecules have a relatively higher refractive index than solvents or air [3] and were used for (bio)sensing applications [4,5,6,7,8,9]. Moreover, gold nanoparticles (Au NPs) were specifically biofunctionalized [10,11], as gold has many easily polarizable conduction electrons [12] and high room-temperature stability when compared to the other metals, such as silver [13,14,15]. However, Au NPs have a smaller magnitude of real-part dielectric function compared to other metals such as silver, which results in less polarizing charges in Au NPs when excited by the incidental electromagnetic fields. As a consequence, Au NPs have normally shown an inferior refractive index sensitivity (up to a twofold decrease) compared to that of the same geometric Ag NPs. Therefore, enhancing the plasmonic sensitivity of Au NPs while maintaining the chemical stability plays a critical role in fabricating robust (bio)active LSPR platforms [16].

Several studies report using different types of composed metallic/non-metallic NPs for increasing the LSPR sensitivity of biosensors [17,18,19,20]. Thus, indium tin oxide (ITO) was used for its conductivity and high optical transmittance, and due to their excellent conductive properties [21,22,23], an array of gold nanoparticles on ITO was used for SERS measurements [24]. Moreover, the plasmonic performance of metallic NPs depends on the chemical compositions and physical dimensions of the metallic nanoparticles [25,26].

Presently, the gold nanoparticles are fabricated by using either “bottom-up” chemical or “top-down” lithographical methods [27]. For instance, the vacuum evaporation of gold thin film on a flat substrate and annealed at a high temperature is considered as a simple and cost-effective approach to obtain stable and tunable gold plasmonic nanostructures of different sizes [28,29,30,31].

The present work focuses on the fabrication of stable gold/ITO-nanostructures on glass coverslips when using different ITO and Au thicknesses and two annealing protocols (referred to as one-step and two-step protocols), in order to conclude on the best optical (plasmonic sensitivity) and electrical performances of hybrid Au/ITO-nanostructures. A proof-of-concept LSPR sensing of a Raman-model molecule, 1,2-bis-(4-pyridyl)-ethene (BPE) in aqueous solution, on annealed ITO/gold-nanostructures embedded in glass coverslips, is reported.

## 2. Materials and Methods

### 2.1. Materials

Round-shaped glass coverslips of 11 mm in diameter (Carl Roth GmbH + Co., KG Karlsruhe, Germany,) were used for the evaporation of thin Au and ITO/Au films. Before the evaporation, coverslips were cleaned using Decon 90 (Decon Laboratories^TM^ Decon 90^TM^) liquid detergent (Fisher Scientific, Göteborg, Sweden) and rinsed with ultrapure water (18.2 MΩ cm) produced by a Millipore Milli-Q water purification system (Molsheim, France). The 1,2-bis-(4-ppyridyl)-ethene (BPE) molecule was purchased from Sigma-Aldrich (Schnelldorf, Germany).

### 2.2. Cleaning Procedure of Coverslips

Coverslips were degreased with Millopore distilled water and a detergent solution (Decon 90) (ratio 2:8, *v*/*v*) in an ultrasonic distilled water bath (Elmasonic S30H model, Elma Schmidbauer GmbH, Singen, Germany) at 50 °C for 15 min. The resulted samples were rinsed with an excess amount of deionized water, dried under an N_2_ stream, and were subjected to another ultrasonication washing in deionized water at 50 °C for 5 min, with the process repeated three times. Finally, the glass substrates were rinsed three times with deionized water, dried in an oven at 100 for 10 min and prepared for the gold evaporation step.

### 2.3. Evaporation of Gold and ITO on Glass Coverslips

**Figure 1:**
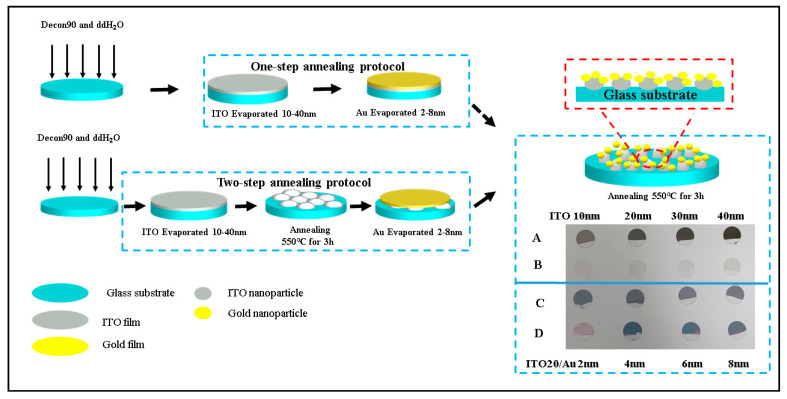
Evaporation and annealing of different indium tin oxide (ITO) thicknesses on round glass coverslips by either the one-step or two-step annealing protocol coated with ITO-20 nm/Au-(2–8 nm) nanoparticles (NPs).

The gold (purity: 99.99%, NEYCO company, Vanves, France) and ITO (In_2_O_3_/SnO_2_, 90/10 wt%, ITO) (Kurt J. Lesker company, Jefferson Hills, PA, USA) evaporation steps were conducted in an evaporator (MEB 400, PLASSYS, Marolles-en-Hurepoix, France) using the electron beam evaporation mode at ambient temperature under a high vacuum (pressure was around 1.0 × 10^−6^ Torr). The round glass coverslips were labeled with a scotch band on an external site for correct handling, fixed on a circular evaporation plate (200 mm diameter) and finally exposed to gold or ITO vapors in the evaporator (Figure 1). The evaporating rate was adjusted to ~0.01 nm/s by slowly changing the working current intensity. The evaporated film thickness was monitored with a built-in quartz crystal sensor.

### 2.4. Annealing of Gold- and ITO-Coated Coverslips

**Figure 2:**
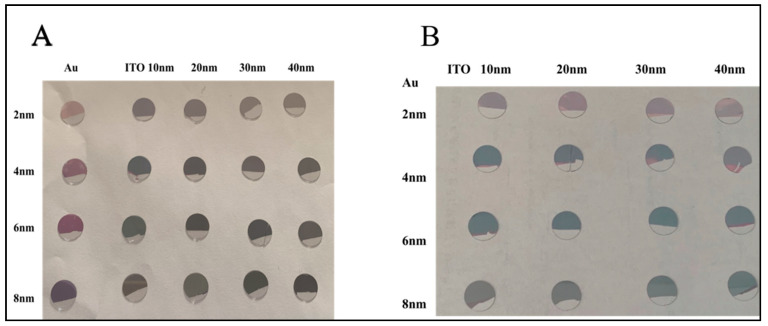
Round glass coverslips coated with Au and ITO-Au thin films and exposed for 3 h at 550 °C. (**A**) *One-step annealing protocol* and (**B**) *Two-step annealing protocol*.

One-step thermal annealing: After the glass substrate evaporation with different ITO and Au thicknesses, the modified glass samples were transferred onto a high-temperature hot plate to conduct the thermal annealing in the presence of oxygen at 550 °C for 3 h (Figure 2A).

Two-step thermal annealing: First, the glass substrates were coated with different ITO thicknesses and annealed immediately at 550 °C for 3 h. Second, annealed glass substrates were exposed to different thicknesses of gold. After the evaporation, the modified Au/ITO/round glass coverslips were transferred to a high-temperature oven or onto a hot plate to conduct the thermal annealing in the presence of oxygen at 550 °C for 3 h (Figure 2B) for the second time.

### 2.5. Instruments

Round -shaped glass coverslips coated with ITO/Au NPs were characterized through several techniques: (i) scanning electron microscope (SEM, FEG-SU8030, Tokyo, Japan), (ii) energy-dispersive X-ray spectroscopy (EDS) (Thermo Fisher Scientific, Waltham, MA, USA) using a field emission scanning electron microscope (FE-SEM, Hitachi SU8030, Hitachi, Ltd, Tokyo, Japan) equipped with a Thermo Scientific Ultradry X-ray detector and (iii) atomic force microscope (AFM).

Moreover, the EDS spectra were recorded and analyzed using the NORAN System 7 software from Thermo Fisher Scientific. The acceleration voltage and working distance were 7 KV and 15 mm, respectively. Atomic Force Microscope (AFM, Bruker ICON, Billeric, MA, USA) with a cantilever ScanAsyst-Air in silicon nitride with a tip height of 2.5–8.0 nm. A spring constant of 4 N/m and a reflective aluminum coating on the backside in standard ScanAsyst-Air mode were used to characterize the morphology of Au or ITO/Au NPs. The Ohm tester (LOMVUM TRMS 6000, Germany) was used for measuring the conductivity of the samples.

An inhouse-built confocal extinction measurement system was also used to record the LSPR spectra from nanochips in the UV-Vis-near-IR (UV-Vis-NIR) ranging from 200 to 1000 nm. For LSPR platform investigations, a 1,2-bis-(4-ppyridyl)-ethene (BPE) molecule was purchased from Sigma-Aldrich (Schnelldorf, Germany).

### 2.6. Preparation of BPE Solutions

Three BPE concentrations (10^−7^, 10^−9^ and 10^−12^ M) were prepared from a stock solution (97% (*v*/*v*) BPE).

### 2.7. Statistical Analysis

SEM images were analyzed using Public Domain ImageJ software, while AFM images (1 μm × 1 μm) were analyzed using Gwyddion. Ra and RMS parameters were used to estimate the surface roughness of the annealed nanostructured coverslips. The experimental data were treated using the GraphPad prism software.

## 3. Results

### 3.1. Optimization of the Annealing Process of Au/ITO Coated Coverslips

**Figure 3:**
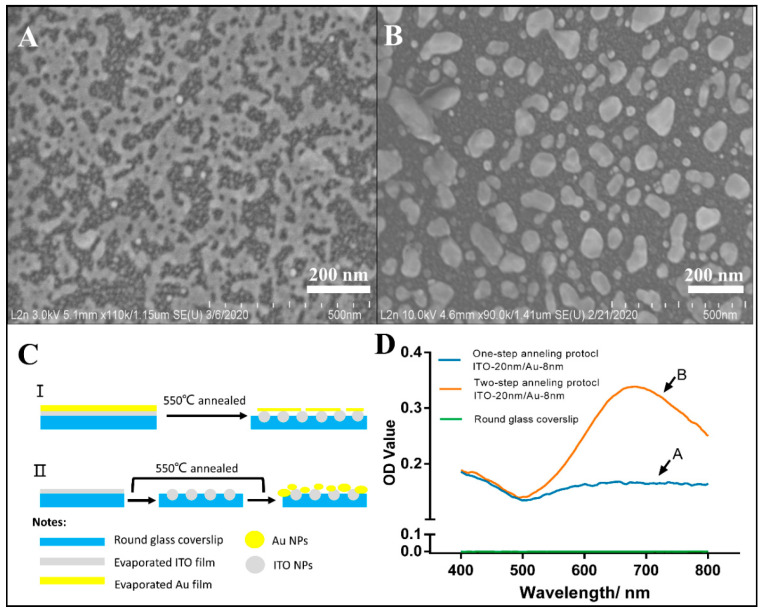
SEM image of (**A**) one-step and (**B**) two-step annealed ITO-20 nm/Au-8 nm NPs; Nanostructuration of coverslips with ITO-20 nm/Au-8 nm NPs (**C**) and the localized surface plasmon resonance (LSPR) characterization of one-step and two-step annealed ITO-20 nm/Au-8 nm NPs samples (**D**).

One-step and two-step annealing processes have been studied to develop the active plasmonic nanostructured surfaces on tiny coverslips (Figure 3C).

Figure 3A,B depicts the SEM morphology of the evaporated Au-8 nm on ITO-20 nm after one-step and two-step annealing protocols. They clearly show that, in the first case, a percolated film was observed, whereas in the second case, well-formed independent nanostructures were obtained. Previous experiments of gold thin films evaporated on glass coverslips and annealed in the same conditions led to well-formed nanoparticles, as was observed in the two-step annealing process used in this paper. This shows that the first annealing of ITO before the gold evaporates produces surfaces with a dilation coefficient similar to that of the glass coverslip, whereas the one-step annealing of ITO and gold does not. The difference in the thermomechanical properties explains the difference in the observed microstructure.

Additionally, several SEM images of different thicknesses of evaporated ITO and Au film, fabricated by both one-step and two-step annealed protocols, are shown in Figures S1 and S2.

Moreover, EDS measurements of annealed substrates confirm the presence of Au, In and Sn elements (inset of Figure S3) with a pronounced LSPR peak (e.g., 681 nm with an optical density value of 0.34) for ITO 20 nm/Au 8 nm NPs, while in the case of one-step annealed glass, no LSPR peak is recorded (Figure 3D).

This different optical behavior is in good agreement with the structure of the film as seen by SEM characterization. As expected, only well-formed nanoparticles led to plasmonic resonance.

For comparison, the EDS spectra of ITO-10 and ITO 40 nm coating coverslips before and after annealing are reported in Figure S4.

Therefore, in the case of optical detection, the two-step annealing process is considered the optimized route for large-scale fabrication of nanoparticles on solid supports.

#### Electrical Measurements

The conductive nature of the two types of annealed surfaces is also investigated for further use in electrochemical measurements.

**Figure 4:**
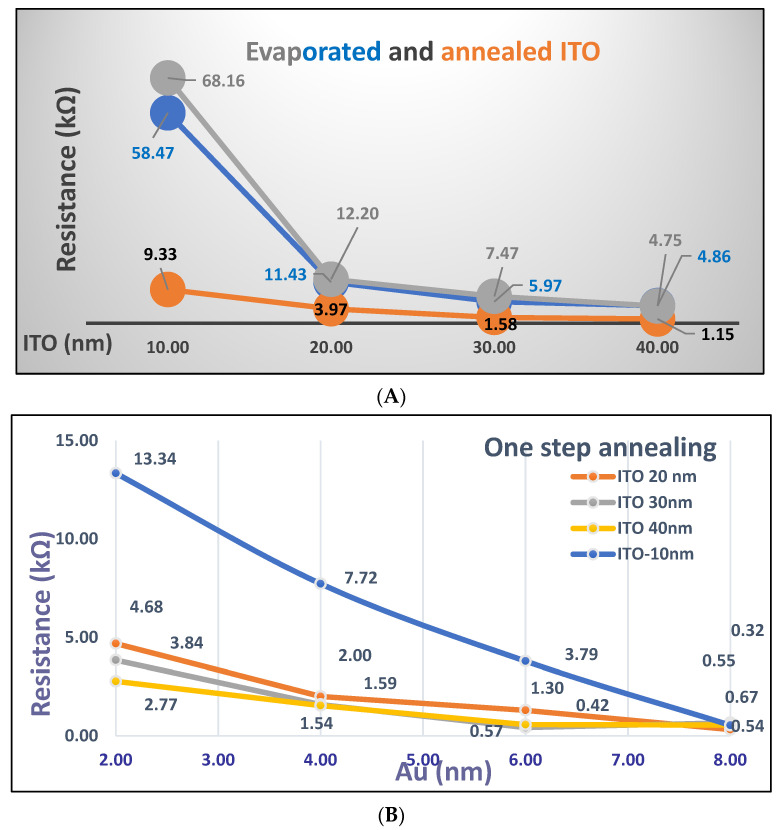

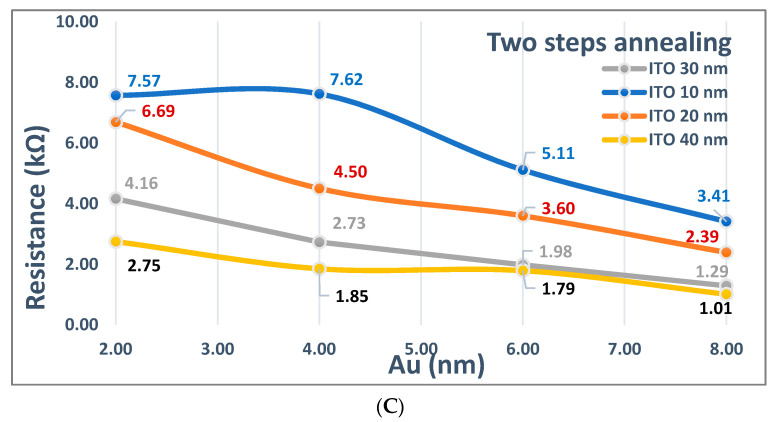
Resistance variation versus (i) ITO thicknesses before and after one-step and two-step annealing processes (**A**), (ii) Au thickness after one-step annealing (**B**) and Au thickness after two-step annealing (**C**) at 500 °C.

Figure 4A depicts the resistance variation in the deposited ITO film on two coverslips versus four ITO thicknesses (10, 20, 30 and 40 nm) before (in grey and blue) and after annealing at 500 °C. The resistance values of one-step annealed coverslips are shown in the brown color, while the second commercial coverslip was used for the two-step annealing protocol.

As is usually observed, before annealing, the resistance values decrease when the thickness increases from 10 to 40 nm, probably due to a homogenate ITO covering of the glass surface. Thus, a strong decrease in resistance is observed for deposited ITO 20, 30 and 40 nm when compared with ITO 10 nm. Moreover, before annealing, the two coverslips samples used for one-step and two-step annealing protocols exhibit very similar behavior with very similar values of resistance. It is noticed that the annealing process enhances the electrical conductivity, especially for the lowest deposited film of ITO.

Figure 4B,C depicts the resistance variation in annealed ITO/Au coverslips coated with different ITO/Au films when using the one-step and two-step annealing protocols. Similar behaviors are observed for both thermal treatments with a decrease in the resistance values versus increases in ITO and Au thicknesses from 10 to 40 nm and from 2 to 8 nm, respectively. Moreover, the resistance values are always higher at a given gold thickness and for the lowest ITO thicknesses.

In the case of the one-step annealing of ITO 10 nm/Au 2, 4, 6 and 8 nm, the resistance values show an abrupt decrease when compared with those obtained using the two-step- treatments, for which a certain homogeneity of the dispersion of the resistance values for different ITO/Au is observed.

For instance, the resistance of the annealed 10 nm ITO-coated coverslip (9.3” kΩ, Figure 4A) is higher than the resistance obtained for the two-step annealing coverslip coated with 10 nm ITO and 2 nm (7 KΩ, Figure 4C). Such dispersion of the resistance values can be attributed to the gold nanoparticle formation that enabled dual optical and electrochemical detections on the same annealed nanostructured coverslip. Therefore, the following section will focus on the characterization of two-step annealing Au/ITO-coated coverslips and their use in sensitive chemosensing.

### 3.2. AFM Characterization of Two-Steps Annealed Glass Surfaces

**Figure 5:**
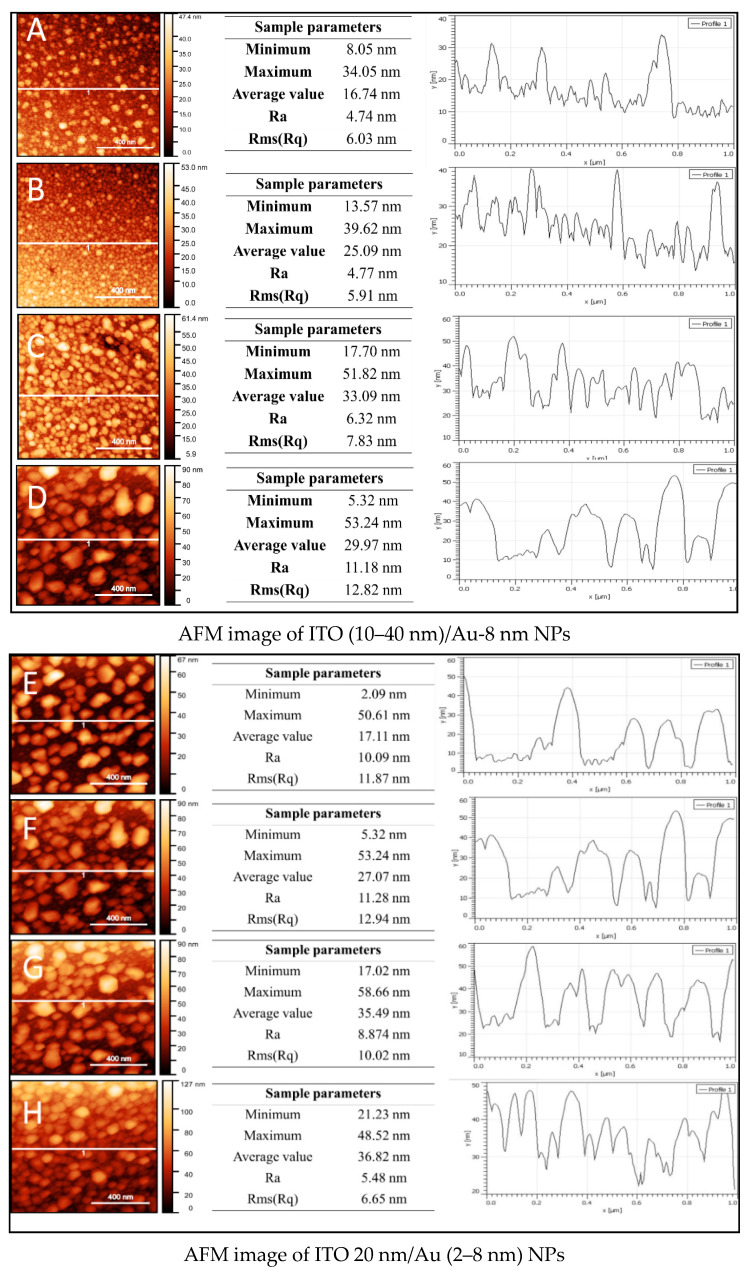
Atomic force microscopy (AFM) images of coverslips coated with (i) different ITO thicknesses (**A**) 10 nm, (**B**) 20 nm, (**C**) 30 nm and (**D**) 40 nm followed by 8 nm Au thickness and (ii): 20 nm ITO further coated with (**E**) Au-2 nm NPs, (**F**) Au-4 nm NPs, (**G**) Au-6 nm NPs, (**H**) Au-8 nm.

The surface roughness of two-step annealed coverslips coated with ITO (10–40 nm) and Au-8 nm, named Configuration 1, was estimated through AFM imaging and height profile analysis (Figure 5A–D). The same measurements were carried out for 20 nm ITO and Au thickness varying from 2 to 8 nm named Configuration 2 (Figure 5E–H).

**Figure 6:**
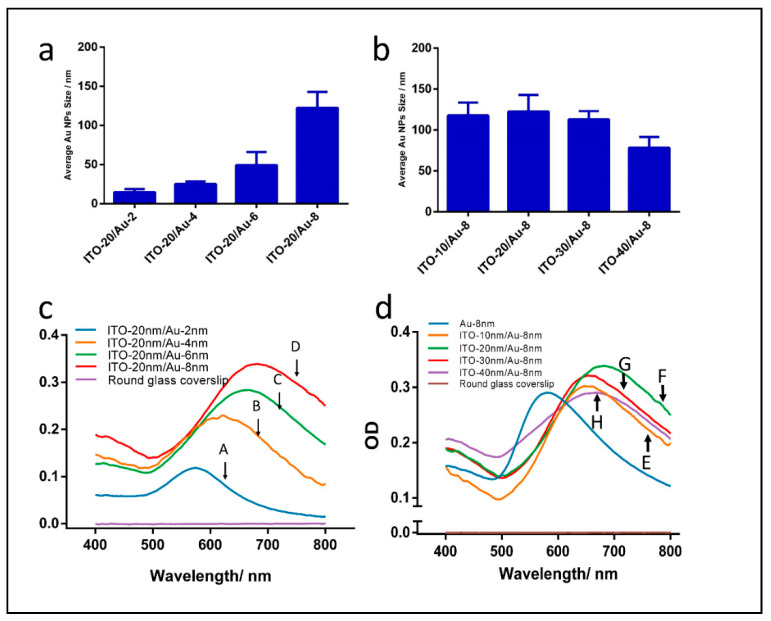
Estimation of nanoparticles sizes after two-step annealing protocol at 550 °C and their LSPR behavior in air-sensing conditions: (**a**,**c**) using annealed 20 nm ITO/(2–8 nm Au) and (**b**,**d**) (10–40 nm ITO)/8 nm Au coatings on coverslips.

In the case of Configuration 1, the roughness values increased relative to the thickness of the ITO and can be adjusted by a quadratic function (R^2^ = 0.997), whereas for Configuration 2, the roughness values vary slightly for samples from 2 to 4 nm Au and decrease for samples from 6 to 8 nm Au. In fact, the decrease in the roughness values is correlated with the increase in the size of nanoparticles (Figure 6), the latter being controlled by the thickness of gold and ITO.

During the two-step annealing process, the evolution of the nanocomposite structures was divided into two main steps. In the first step, the ITO thin films of different thicknesses were evaporated on coverslips and heated at 550 °C. During the heating treatment, the dewetting of thin films started with the formation of voids, which could take place at the grain boundaries, the film–substrate interface and especially in areas of high local stress. Subsequently, the formed voids grew larger, leading to the break-up of continuous film into droplets or islands. In the second step, the gold film, 8 nm in thickness, was subsequently evaporated over annealed ITO NPs formed on coverslips.

### 3.3. Optical Properties of Two-Steps Annealed Surfaces

#### 3.3.1. Color Variation of the Coverslip Surface Versus Evaporated Gold Thickness

After the independent annealing of 2, 4, 6 and 8 nm Au evaporated on four annealed 20 nm ITO-coated coverslips, colors (Figure 2) from violet (2 nm Au) to light blue (4 and 6 nm Au) or dark blue (8 nm Au) was obtained. Further, NP sizes (Figure 6a) were estimated as logical increases, with increases in Au thicknesses from 2 to 8 nm, which correspond to the color variation. Moreover, for ITO-40 nm/Au-8 nm annealed coverslips, the average NPs size was much smaller than the other three nanostructuration configurations (Figure 6b).

#### 3.3.2. LSPR Characterization

By using different nominative Au thicknesses, large-scale and tunable resonant wavelengths and maximum optical density properties of annealed ITO/Au nanostructures formed on tiny coverslips were achieved. In summary, a correlation between the initial ITO/Au film thickness and their plasmonic properties are reported in Table 1 and plotted in Figure 6c,d.

Among the annealed samples, those with hybrid ITO-20 nm/Au-8 nm show the most intensive plasmonic properties in terms of the highest OD at 0.342 a.u. and λmax at 669 nm when compared with naked Au coverslips (OD of 0.290 a.u. and λmax at 581 nm) (Figure 6c). Moreover, as the initial evaporated gold film thicknesses increase, the peak amplitude and resonant wavelength clearly increase. For instance, when the Au thin film thickness increased from 2 to 8 nm, the resonant wavelength of ITO/Au NPs red-shifted 31.42, 76.32, 82.90 and 90.24 nm, compared with those of Au NPs.

With this concern, an optimal 8 nm Au coating configuration on different annealed ITO thicknesses is herein proposed. Furthermore, based on the morphological information of resulted nanoparticles, the maximum optical value is higher than that of the monometallic Au NPs (Figure 6d).

According to the resistance measurements, the lower ohmic losses could be responsible for the better LSPR proprieties of ITO/Au NPs than that of monometallic Au NPs. Moreover, the smaller average size of ITO-20/Au-2 nm NPs may be responsible for the decrease in maximum optical density.

To conclude, coverslips modified with ITO-20 nm/Au-8 nm have the highest plasmonic properties and were selected for the chemosensing application of BPE molecules

### 3.4. LSPR Chemosensing of BPE Traces on Two-Steps ITO/Au Annealed Coverslips

**Figure 7:**
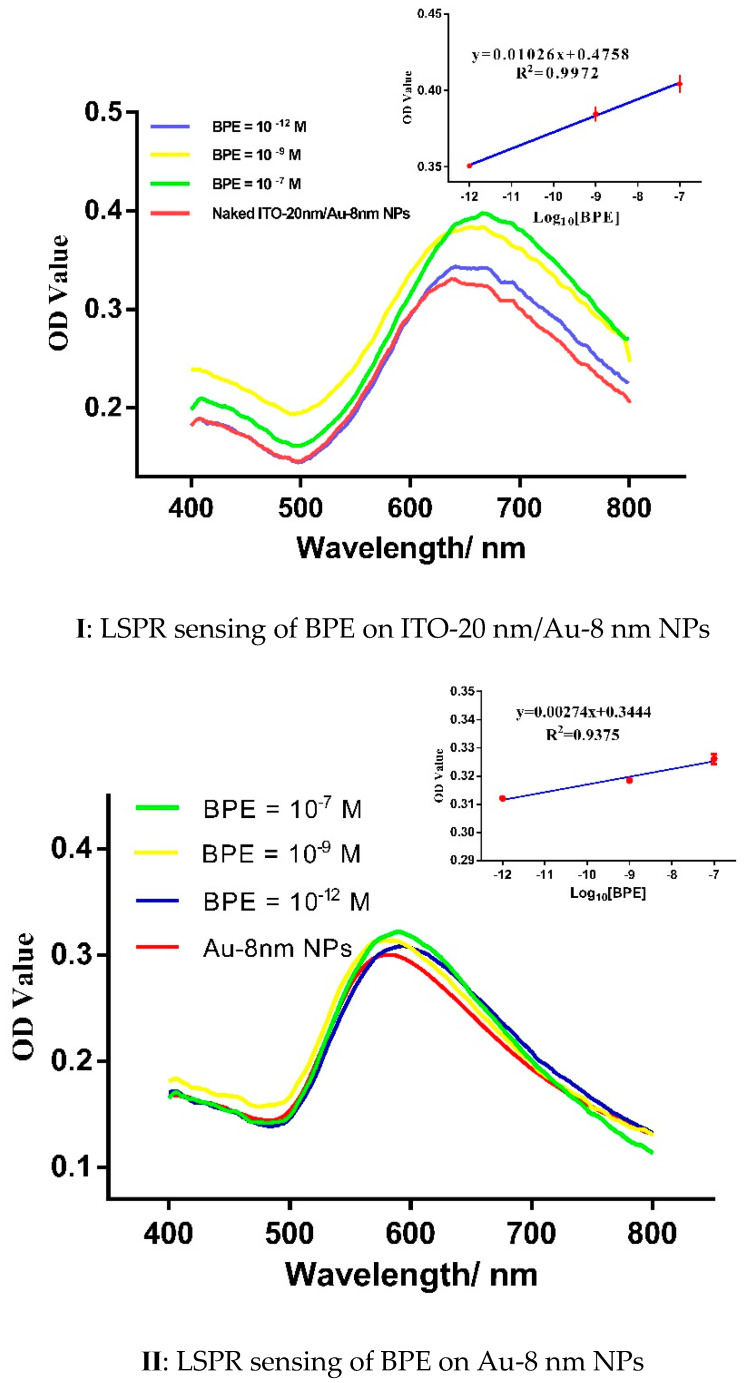
LSPR sensing of BPE traces on annealed (**I**) ITO-20 nm/Au-8 nm NPs and (**II**) Au-8 nm NPs formed on small-sized coverslips. The insets show the linear evolution of OD values for the LSPR tested concentrations on two types of nanostructured substrates.

LSPR spectra from tiny, dried drops of BPE concentrations (10^−7^ M to 10^−12^ M), deposited on either ITO-20 nm/Au-8 nm NPs or Au-8 nm NPs (Figure 7), were recorded and compared. For instance, in the case of 10^−7^ M BPE on Au-8 nm NPs and ITO-20 nm/Au-8 nm NPs, 8 nm red-shift of the resonant wavelength with an increase of 0.023 OD value and a larger red-shift of wavelength (16 nm) with a much larger maximum 0.064 OD value, respectively, were noted. A logical and significant increase in the optical density from 0.355 for 10^−12^ M BPE to 0.385 for 10^−9^ M BPE was recorded on the ITO-20 nm/Au-8 nm NP-coated coverslips.

By using a linear fitting of the LSPR measurements, two linear regression coefficients of 0.9972 and 0.9375 were calculated for ITO-20 nm/Au-8 nm NPs and Au-8 nm NPs. These values also confirm a better plasmonic sensitivity for the ultra-thin supports, based on ITO-20 nm/Au-8 nm NPs (Figure 7, inset).

To conclude, the lowest BPE concentration detected was 10^−12^ M on two-step annealed ITO/Au NPs formed on small, round, glass coverslips.

## 4. Conclusions

To summarize, in this work, large-scale, conductive and annealed ITO/Au nanostructures were fabricated for the first time on small-sized, round, glass coverslips. Two protocols were proposed for the controlled nanostructuration of solid supports. Results have shown that the two-step annealing protocol is the ideal way to fabricate homogeneous ITO/Au NPs onto coverslips at a low cost. Meanwhile, by evaporating films of Au of different nominative thicknesses, large-scale tunable ITO/Au NPs with LSPR properties (tunable resonant wavelength) were achieved, which could be significantly convenient for further sensing applications in either UV-Vis or UV-Vis-IR domains. Moreover, it was found that fabricated ITO/Au NPs have a higher maximum extinction in the visible spectrum range with more sensitive plasmonic responses than those of Au NPs. By using ITO-20 nm/Au-8 nm NPs active LSPR platforms, it is possible to detect 10^−12^ M BPE. Therefore, the proposed nanostructured and conductive substrates can easily be used as sensitive LSPR platforms for biological multiplexing applications.

## Figures and Tables

**Table 1:** LSPR analytical performances of annealed ITO/Au NPs on coverslips.

Type of Substrate	λmax (nm)	Maximum Optical Density	OD_max_/fwhm ^a^ (10^−3^)
ITO-20 nm/Au-2 nm	578	0.119	1.05
ITO-20 nm/Au-4 nm	623	0.233	1.52
ITO-20 nm/Au-6 nm	653	0.285	1.72
ITO-20 nm/Au-8 nm	669	0.342	2.33
ITO-10 nm/Au-8 nm	650	0.307	2.19
ITO-20 nm/Au-8 nm	653	0.342	2.33
ITO-30 nm/Au-8 nm	650	0.323	2.29
ITO-40 nm/Au-8 nm	648	0.291	1.58
Au-8 nm	581	0.290	2.20

^a^ fwhm is the full width at half maximum.

## Supplementary Materials

The following are available online at https://www.mdpi.com/2072-666X/12/3/275/s1, Figure S1: SEM images of one-step (**A**) and two-step (**B**) annealing of round glass coverslips coated with (**A**–**D**) ITO-10 nm/Au NPs (2–8 nm), (**E**–**H**) ITO-20 nm/Au NPs (2–8 nm), (**I**–**L**) ITO-30 nm/Au NPs (2–8 nm) and (**M**–**P**) ITO-40 nm/Au NPs (2–8 nm). The green square and red square show the morphology of optimized surface (**H**) for sensing, Figure S2: SEM images of two-step annealing of coverslips coated with (**A**–**D**) ITO-10 nm/Au NPs (2–8 nm), (**E**–**H**) ITO-20 nm/Au NPs (2–8 nm), (**I**–**L**) ITO-30 nm/Au NPs (2–8 nm) and (**M**–**P**) ITO-40 nm/Au NPs (2–8 nm). Red square shows the morphology of optimized surface ITO 20 nm/Au 8 nm (**H**) for sensing, Figure S3: Energy-dispersive X-ray spectrum and SEM image of the one-step annealed ITO-20 nm/Au-8 nm NPs (**A**) and two-step annealed ITO-20 nm/Au-8 nm NPs (**B**). (inset EDS 2 D mapping of different elements in the ITO/Au NPs.), Figure S4: Energy-dispersive X-ray spectrum and SEM image of coverslips coated with ITO-10 nm before (**A**) and after annealing (**B**) and of coverslips coated with ITO-40 nm before (**C**) and after annealing at 550 °C (**D**).
