# Supplementary material for: Chemosensing on Miniaturized Plasmonic Substrates
Source: Micromachines (Basel). 2021 Mar 6;12(3):275. doi: 10.3390/mi12030275 (PMC8001780; doi:10.3390/mi12030275)
Supplement: Supplementary file 1 [file micromachines-12-00275-s001.pdf]

Supplementary Materials: Chemosensing on Miniaturized Plasmonic Substrates

Pengcheng Wang and Rodica Elena Ionescu

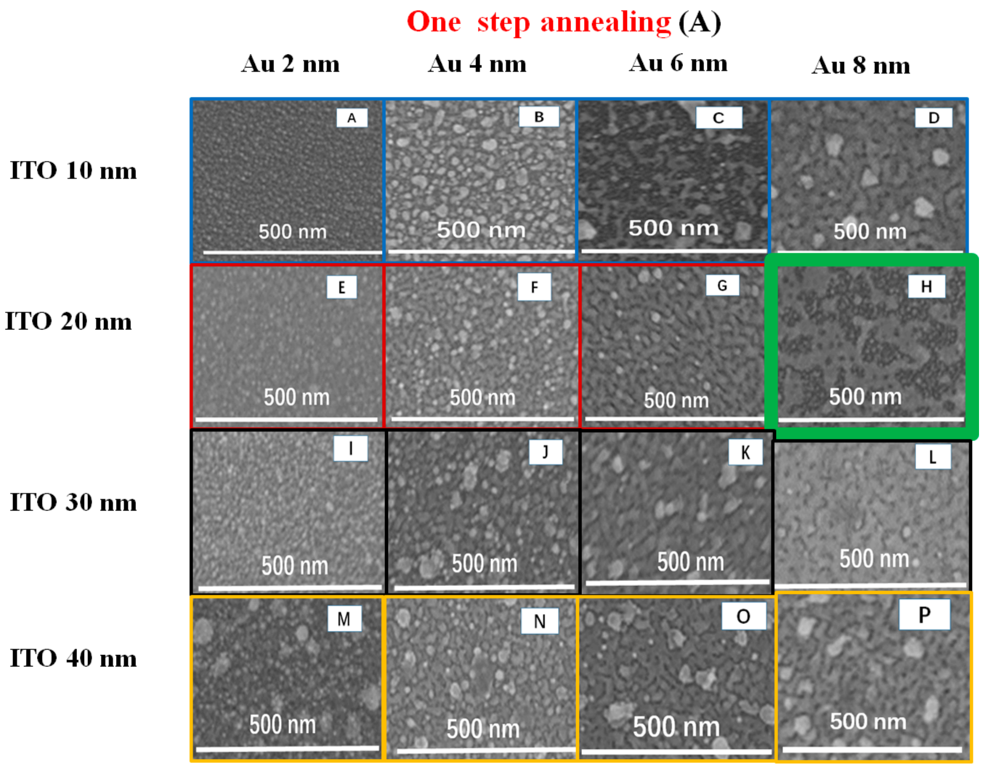

Figure S1. SEM images of one step (**A**) and two steps (**B**) annealing of round-shaped glass coverslips coated with (**A–D**) ITO-10nm/Au NPs (2–8 nm), (**E–H**) ITO-20nm/Au NPs (2–8 nm), (**I–L**) ITO-30nm/Au NPs (2–8 nm) and (**M–P**) ITO-40nm/Au NPs (2–8 nm). Green square and red square show-he morphology of optimized surface (**H**) for sensing.

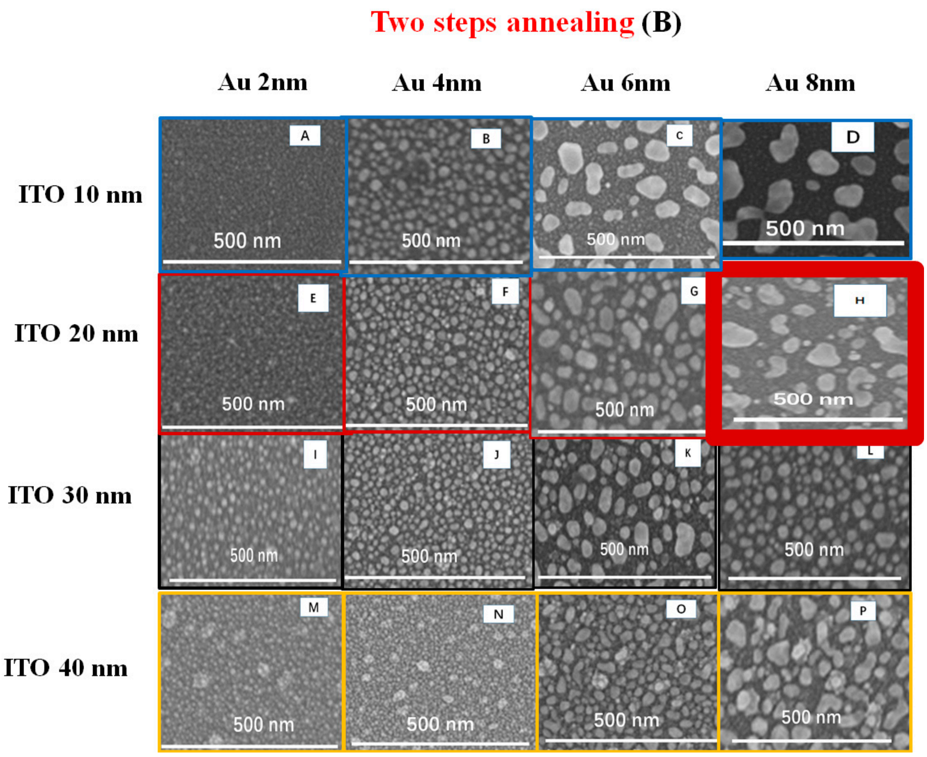

Figure S2. SEM images of two steps annealing of coverslips coated with (**A**–**D**) ITO-10nm/Au NPs (2–8 nm), (**E**–**H**) ITO-20nm/Au NPs (2–8 nm), (**I**–**L**) ITO-30nm/Au NPs (2–8 nm) and (M–P) ITO-40nm/Au NPs (2–8 nm). Red square -the morphology of optimized surface ITO 20nm/ Au 8 nm (**H**) for sensing.

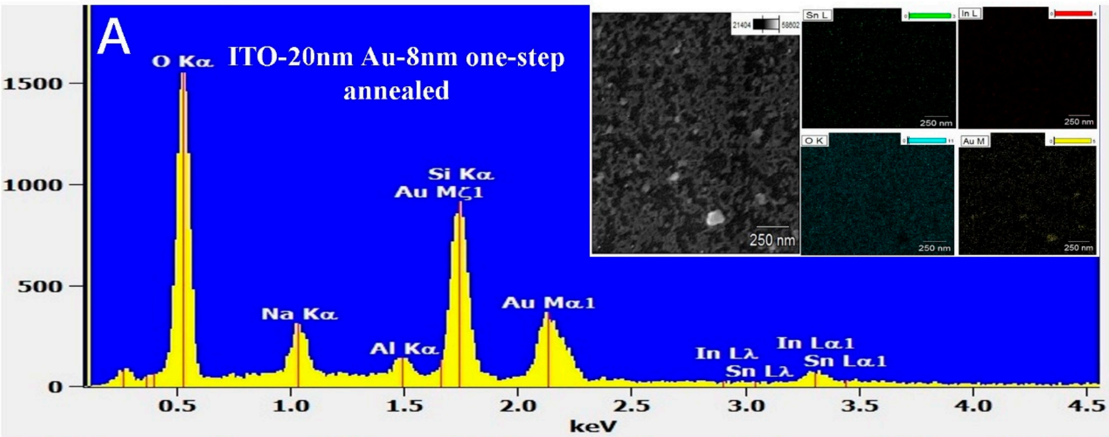

Element Line	Net Counts	Weight %	Weight % Error	Atom %	Atom % Error	Formula	Compnd %
O K	9431	30.2	± 0.3	62.5	± 1.3	O	30.2
Na K	1936	3.7	± 0.1	5.3	± 0.4	Na	3.7
Al K	796	1.5	± 0.1	1.8	± 0.2	Al	1.5
Si K	8026	17.1	± 0.2	20.2	± 0.4	Si	17.1
In L	1386	17.7	± 2.0	5.1	± 1.1	In	17.7
Sn L	30	0.5	± 1.2	0.1	± 0.7	Sn	0.5
Au M	5060	29.4	± 0.6	4.9	± 0.2	Au	29.4
Total		100.0		100.0			100.0

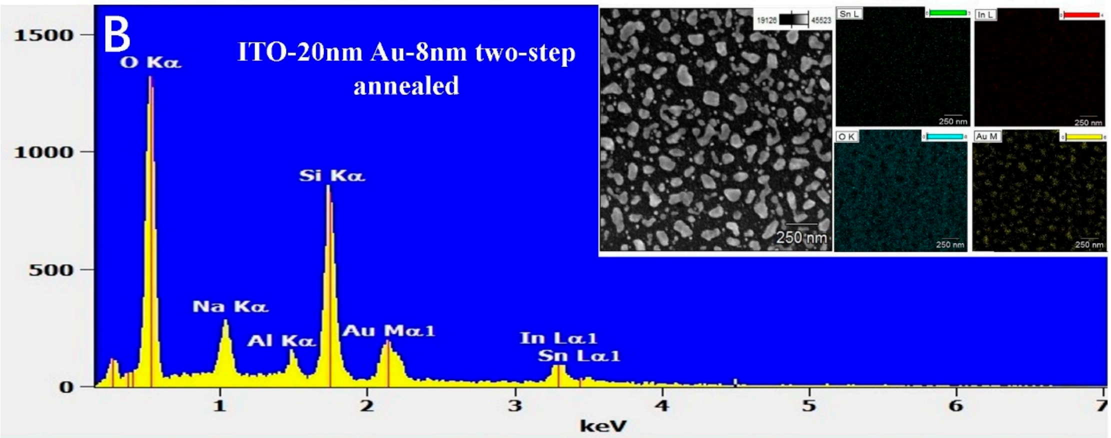

Element Line	*Net Counts*	*Weight %*	*Weight % Error*	*Atom %*	*Atom % Error*	*Formula*	*Compnd %*
O K	8000	31.4	± 0.4	61.7	± 1.4	O	31.4
Na K	1796	4.1	± 0.1	5.5	± 0.4	Na	4.1
Al K	770	1.7	± 0.1	2.0	± 0.2	Al	1.7
Si K	7365	18.6	± 0.2	20.8	± 0.5	Si	18.6
In L	1724	25.8	± 2.0	7.1	± 1.1	In	25.8
Sn L	0	0.0	---	0.0	± 0.0	Sn	0.0
Au M	2675	18.4	± 0.5	2.9	± 0.2	Au	18.4
Total		100.0		100.0			100.0

Figure S3. Energy-dispersive X-ray spectrum and SEM image of the one-step annealed ITO-20nm/Au-8nm NPs (**A**) and two-step annealed ITO-20nm/Au-8nm NPs (**B**). (Inset: EDS 2D mapping of different elements in the ITO/Au NPs.)

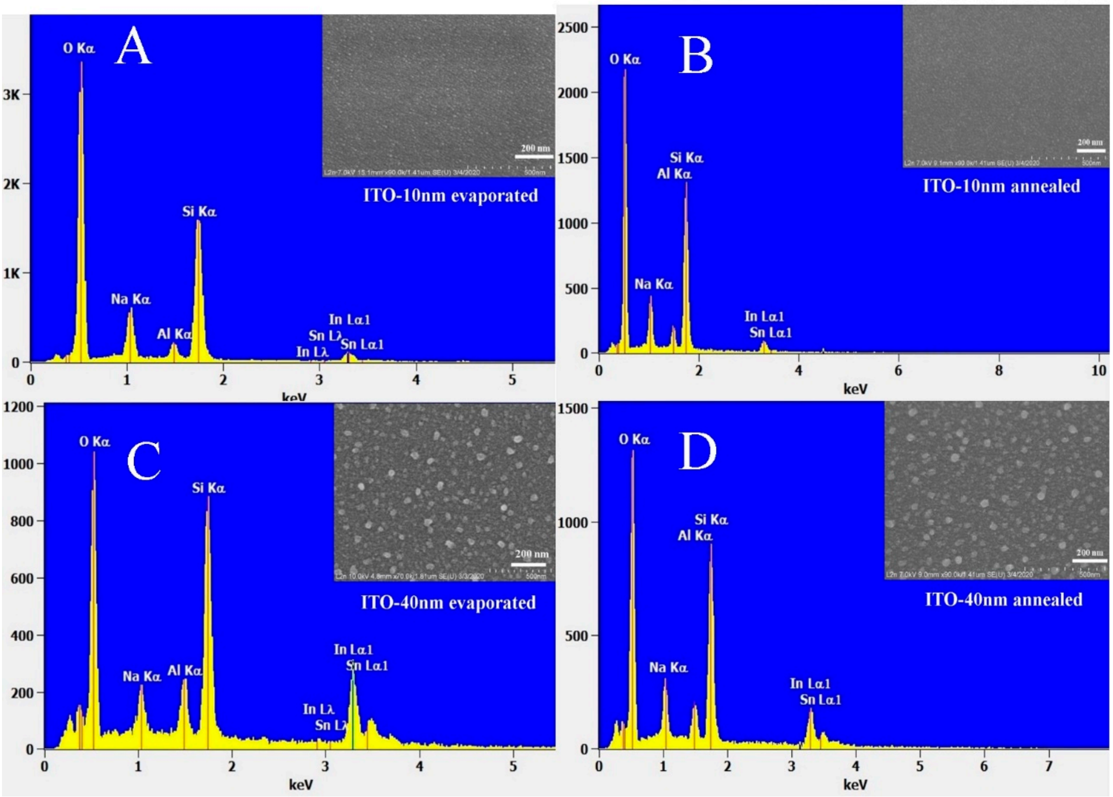

Figure S4. Energy-dispersive X-ray spectrum and SEM image of coverslips coated with ITO-10 nm before (**A**) and after annealing (**B**) and of coverslips coated with ITO-40 nm before (**C**) and after annealing at 550 °C (**D**).
